# Supplementary material for: Getting More Out of Biomedical Documents with GATE's Full Lifecycle Open Source Text Analytics
Source: PLoS Comput Biol. 2013 Feb 7;9(2):e1002854. doi: 10.1371/journal.pcbi.1002854 (PMC3567135; doi:10.1371/journal.pcbi.1002854)
Supplement: Dataset S1 — GATE software. Dataset S1 bundled with this paper contains a distribution of GATE (or see http://gate.ac.uk/download/). (TGZ) [file pcbi.1002854.s001.tgz › plos-gate/plugins/Tagger_MetaMap/doc/gate/metamap/TaggerMode.html]

TaggerMode


---


|  |  |  |
| --- | --- | --- |
| **Overview** \| **Package** \| **Class** \| **Use** \| **Tree** \| **Deprecated** \| **Index** \| **Help** | |  |
| **PREV CLASS**   NEXT CLASS | **FRAMES**    **NO FRAMES**     **All Classes** |  |
| SUMMARY: NESTED \| ENUM CONSTANTS \| FIELD \| METHOD | DETAIL: ENUM CONSTANTS \| FIELD \| METHOD |  |


---


## gate.metamap Enum TaggerMode

```
java.lang.Object
  java.lang.Enum<TaggerMode>
      gate.metamap.TaggerMode
```

**All Implemented Interfaces:**: java.io.Serializable, java.lang.Comparable<TaggerMode>

---

``` public enum TaggerMode extends java.lang.Enum<TaggerMode> ```

---

**Enum Constant Summary**

`AllOccurrences`

`CoReference`

`FirstOccurrenceOnly`


| **Method Summary** | |
| --- | --- |
| `static TaggerMode` | `valueOf(java.lang.String name)`             Returns the enum constant of this type with the specified name. |
| `static TaggerMode[]` | `values()`             Returns an array containing the constants of this enum type, in the order they are declared. |

**Methods inherited from class java.lang.Enum**

`clone, compareTo, equals, finalize, getDeclaringClass, hashCode, name, ordinal, toString, valueOf`

**Methods inherited from class java.lang.Object**

`getClass, notify, notifyAll, wait, wait, wait`

**Enum Constant Detail**

### FirstOccurrenceOnly

```
public static final TaggerMode FirstOccurrenceOnly
```

---


### CoReference

```
public static final TaggerMode CoReference
```

---


### AllOccurrences

```
public static final TaggerMode AllOccurrences
```


**Method Detail**

### values

```
public static TaggerMode[] values()
```

:   Returns an array containing the constants of this enum type, in
    the order they are declared. This method may be used to iterate
    over the constants as follows:

    ```
    for (TaggerMode c : TaggerMode.values())
        System.out.println(c);
    ```

    :   **Returns:**: an array containing the constants of this enum type, in the order they are declared

---


### valueOf

```
public static TaggerMode valueOf(java.lang.String name)
```

:   Returns the enum constant of this type with the specified name.
    The string must match *exactly* an identifier used to declare an
    enum constant in this type. (Extraneous whitespace characters are
    not permitted.)

    :   **Parameters:**: `name` - the name of the enum constant to be returned. **Returns:**: the enum constant with the specified name **Throws:**: `java.lang.IllegalArgumentException` - if this enum type has no constant with the specified name: `java.lang.NullPointerException` - if the argument is null


---


|  |  |  |
| --- | --- | --- |
| **Overview** \| **Package** \| **Class** \| **Use** \| **Tree** \| **Deprecated** \| **Index** \| **Help** | |  |
| **PREV CLASS**   NEXT CLASS | **FRAMES**    **NO FRAMES**     **All Classes** |  |
| SUMMARY: NESTED \| ENUM CONSTANTS \| FIELD \| METHOD | DETAIL: ENUM CONSTANTS \| FIELD \| METHOD |  |


---
